# GnRH Peptide Antagonist: Comparative Analysis of Chemistry and Formulation with Implications for Clinical Safety and Efficacy

**DOI:** 10.3390/ph18010036

**Published:** 2024-12-31

**Authors:** Shikha Patel, Bhagawati Saxena, Priti Mehta, Sarfaraz K. Niazi

**Affiliations:** 1 Department of Pharmaceutical Analysis, Institute of Pharmacy, Nirma University, Ahmedabad 382481, India; 20ptphdp126@nirmauni.ac.in (S.P.); drpritimehta@nirmauni.ac.in (P.M.); 2 Department of Pharmacology, Institute of Pharmacy, Nirma University, Ahmedabad 382481, India; bhagawati.saxena@nirmauni.ac.in; 3 College of Pharmacy, University of Illinois, Chicago, IL 60612, USA

**Keywords:** GnRH peptide antagonists, assisted reproductive technologies, prostate cancer, Teverelix, Degarelix, Ganirelix, Cetrorelix, Abarelix

## Abstract

Overexpression of the gonadotropin-releasing hormone receptor (GnRH-R) plays a vital role in the advancement of reproductive malignancies such as ovarian, endometrial, and prostate cancer. Peptidomimetic GnRH antagonists are a substantial therapeutic development, providing fast and reversible suppression of gonadotropins by directly blocking GnRH-R. Unlike typical GnRH agonists, these antagonists prevent the early hormonal flare, have a faster onset of action, and have a lower risk of cardiovascular problems. These characteristics qualify GnRH antagonists as revolutionary therapy for diseases such as advanced prostate cancer, endometriosis, uterine fibroids, and in vitro fertilization procedures. Key GnRH peptide antagonists authorized by the regulatory agencies include Cetrorelix, Ganirelix, Abarelix, Degarelix, and Teverelix. Assisted reproductive technologies (ART) are dominated by Cetrorelix and Ganirelix, while Degarelix and Abarelix have shown significant promise in treating advanced prostate cancer. Teverelix appears as a next-generation GnRH antagonist with an ideal mix of efficacy and safety, showing promise in a variety of reproductive and hormone-dependent illnesses. This review investigates the pharmacological role of GnRH in reproductive physiology and its consequences in disease, emphasizing structural advances in third- and fourth-generation GnRH antagonists. All GnRH peptide-based antagonists were analyzed in detail for formulation strategy, pharmacokinetics, effectiveness, and safety. This review also emphasizes GnRH antagonists’ clinical promise, providing insights into their evolution and the possibility for future research in developing safer, more effective treatments for complicated hormonal diseases.

## 1. Introduction

Gonadotropin-releasing hormone (GnRH) is an essential regulator of the hypothalamic–pituitary–gonadal (HPG) axis. GnRH regulates reproductive function by inducing the pituitary gland to discharge follicle-stimulating hormone (FSH), as well as luteinizing hormone (LH), which impacts gonadal steroidogenesis and gametogenesis [1,2,3,4]. Recognizing the therapeutic potential of manipulating GnRH structure and function, researchers have developed synthetic peptide analogs. These synthetic GnRH analogs have evolved as indispensable tools for managing various hormone-dependent conditions. Initially, GnRH agonists have dominated the therapeutic landscape, functioning by binding to the GnRH receptor and activating it, which leads to a prolonged release of gonadotropins (FSH and LH). This sustained activation, however, can eventually desensitize the receptor, leading to a decrease in gonadotropin secretion, a process known as receptor downregulation. In contrast, GnRH antagonists work by competitively binding to the GnRH receptor without activating it. This blockage prevents the release of gonadotropins, providing a rapid and reversible inhibition of the HPG axis without the initial surge of hormones that is typically seen with GnRH agonists. This surge, known as the “flare effect”, can be detrimental in certain treatments, making GnRH antagonists a preferred option in some clinical settings.

These GnRH peptide antagonists significantly suppress the production of androgen and estrogen. These drugs are widely used during in vitro fertilization (IVF) cycles for restricted ovarian stimulation [1,5,6]. More recently, they have been approved for use as androgen deprivation therapy (ADT) in advanced prostate cancer [7,8]. Some of the GnRH antagonists are additionally employed to treat benign conditions that respond to hormone suppression, including infertility, uterine fibroids, endometriosis, and precocious puberty [9].

This review aims to comprehensively examine their mechanisms of action and pharmacological properties. We will delve into the structural and chemical modifications of approved, discontinued, and investigational peptide antagonists, analyzing their impact on pharmacokinetics, pharmacodynamics, clinical efficacy, and safety profiles. By understanding these relationships, drug designers can aim to accelerate the development of novel GnRH antagonists with improved therapeutic profiles, including enhanced bioavailability, prolonged duration of action, and targeted delivery. This knowledge will be valuable for researchers in diverse fields, enabling them to explore the potential of GnRH antagonists in other hormone-dependent cancers, such as breast, ovarian, endometrial, and uterine fibroids. Furthermore, these insights can inform the design of innovative peptide-based therapeutic formulations, such as those with high drug loading, prolonged duration of action, and sustained release properties, ultimately aiming to reduce side effects and improve overall therapeutic outcomes.

## 2. Methodology

A thorough review of the literature was achieved using many databases, including Cochrane, Google Scholar, Embase, Web of Science, PubMed, and Scopus, without any time restrictions. The search was focused on identifying studies related to GnRH antagonists and their chemical structure, formulation, pharmacokinetics, safety, and efficacy in various clinical applications. The following keywords were used in the search: “GnRH”, “GnRH antagonist”, “reproductive medicines”, “prostate cancer”, “endometriosis”, “uterine fibroids”, “in vitro fertility”, “assisted reproductive technologies”, “Abarelix”, “Cetrorelix”, “Ganirelix”, “Degarelix”, and “Teverelix”. The identified studies were meticulously screened based on predefined inclusion and exclusion criteria to select relevant articles for the review. Inclusion criteria focused on original research articles, clinical trials, and systematic reviews investigating synthetic peptide-based GnRH antagonist drugs. Studies primarily exploring other therapeutic modalities or lacking comprehensive data on drug characteristics were excluded. Data extraction was performed systematically from the included studies to capture essential information regarding GnRH peptide antagonists’ chemical structure, formulation, pharmacokinetics, safety, and efficacy. Extracted data were analyzed to thoroughly summarize the existing state of knowledge about GnRH peptide antagonist drugs. The analysis described the chemistry, formulation strategies, pharmacokinetic profile, safety concerns, and efficacy of marketed and investigational GnRH peptide antagonist drugs. Comparative analysis was conducted whenever possible to identify trends and potential advantages or disadvantages.

## 3. Hypothalamic–Pituitary–Gonadal (HPG) Axis

In humans and other higher animals, reproduction is essential for species preservation and is intricately regulated by the HPG axis. The primary component of this axis is GnRH, a linear decapeptide produced predominantly by hypothalamic neurons [10]. Pulsatile release of GnRH occurs in the hypophyseal portal circulation system, which is essential for the HPG axis to function appropriately. The pulsatile discharge of GnRH is regulated by kisspeptin neurons located in the hypothalamus. Non-pulsatile or continuous GnRH production could result in the pituitary response’s desensitization and reduced gonadotropin output [11]. GnRH flows to the anterior pituitary, interacting with its corresponding GnRH receptor (GnRHR), expressed in gonadotropic cells. GnRH then stimulates the anterior pituitary to secrete LH and FSH. Both gonadotropins are released in asynchronous patterns in reaction to differential GnRH pulse frequencies. These gonadotropins regulate the maturation and function of peripheral reproductive organs (the testis and ovary) required for fertility. These hormones then migrate to the gonads; promote the fabrication of sex steroids (testosterone, estrogen, and progesterone); and assist gametogenesis (oogenesis in females and spermatogenesis in men). The HPG axis is regulated through a feedback mechanism where sex steroids regulate the synthesis of GnRH, FSH, and LH by giving negative feedback to the hypothalamus and pituitary. In certain phases of the female menstrual cycle, estrogen can exert positive feedback, leading to the LH surge necessary for ovulation. Dysregulation of the HPG axis can result in conditions such as hypogonadism, infertility, and polycystic ovary syndrome (PCOS). Understanding the role of GnRH is crucial in studying reproductive physiology and treating associated illnesses [10,12,13].

## 4. GnRH and GnRH Receptors

GnRH serves as the pivotal neuroendocrine initiator of the HPG axis, activating the anterior pituitary to release gonadotropins LH and FSH. In 1971, two cutting-edge scientists, Roger Guillemin and Andrew Schally, extracted GnRH from the mammalian hypothalamus for the first time. They gained recognition in the scientific community for their ground-breaking work in identifying GnRH and comprehending its function as a crucial regulator of reproductive function. In 1977, they shared the Nobel Prize in Physiology or Medicine for their discoveries on “peptide hormone production of the brain” [14,15]. GnRH exerts its effects primarily by binding to and activating GnRHRs. These GnRHRs are a G protein-coupled receptor (GPCR) coupled principally to the Gαq/11 protein. However, the interaction of GnRH and GnRHRs leads to the stimulation of phospholipase Cβ (PLCβ), leading to the hydrolyzes phosphatidylinositol 4,5-bisphosphate (PIP2) to generate diacylglycerol (DAG) and inositol trisphosphate (IP3). DAG initiates downstream phosphorylation events by triggering protein kinase C (PKC). On the other hand, IP3 interacts with its receptors on the endoplasmic reticulum, inducing the discharge of calcium ions (Ca^2+^) into the cytoplasm [16,17,18]. The rise in intracellular Ca^2+^ is a critical event in gonadotropin secretion and the activation of downstream signaling pathways, including calmodulin/Ca^2+^ calmodulin-dependent protein kinase II (CaMKII) and calmodulin/calcineurin/ nuclear factor of activated T cells (NFAT) cascades. In the Calmodulin/Calcineurin/NFAT pathway, Ca^2+^ binds to calmodulin, which activates calcineurin, resulting in the dephosphorylation and stimulation of NFAT. In the calmodulin/CaMKII pathway, Ca^2+^/CaMKII is activated, leading to further phosphorylation events [16,19]. Activation of PKC results in downstream phosphorylation of the jun-N-terminal kinase (JNK), extracellular signal-regulated kinase 1 and 2 (ERK1/2), and mitogen-activated protein kinases (MAPKs). ERK1/2 is involved in regulating gene expression and cell functions. JNK plays a role in stress responses. MAPKs play a pivotal role in numerous cellular functions, which include differentiation, proliferation, and survival.

GnRHRs additionally regulate the Gαs (Gs alpha subunit) pathway, where it couples with Gαs. The stimulation of adenylyl cyclase results in increasing cyclic adenosine monophosphate (cAMP) levels. This triggers the phosphorylation of numerous proteins, including the cAMP response element-binding protein (CREB), activating protein kinase A (PKA), and influencing transcription. The intracellular signaling elicited by GnRH is multifaceted and dependent on the cell-specific context, like differential receptor expression on various cells, coupling with variable receptors (e.g., Gαq/11, Gαs, and Gαi) and crosstalk with other signaling pathways (e.g., growth factor signaling and stress responses). These intricate signaling networks orchestrate the transcriptional regulation of LH and FSH subunits [1,16,20]. Overall, the signaling pathways activated by GnRH are complex and finely tuned to the specific context of the cell type and physiological state. This ensures that the GnRH signal is translated into precise and appropriate cellular responses, crucial for regulating reproductive function.

### 4.1. Chemistry of GnRH

Firstly, the GnRH neuropeptide was extracted from porcine and the ovine hypothalamus [4,21,22]. In vertebrates, over 20 GnRH isoforms have been identified. The first two isoforms of GnRH have important autocrine (e.g., immune cells, GnRH neurons, and prostatic and breast cancer cells) and paracrine (e.g., in gonads and placenta) regulatory functions in humans [23,24]. Type I GnRH (GnRH-I) is the initial type of GnRH. GnRH-I is a decapeptide with amino acid sequence pGlu-His-Trp-Ser-Tyr-Gly-Leu-Arg-Pro-Gly-NH2 vital for reproduction. Its primary site of synthesis and release is from neurons in the hypothalamus. Originally isolated from the chicken brain, type II GnRH (GnRH-II) is the second form of GnRH. GnRH-II is 70% similar to GnRH-I and differs from it for the His5Trp7 and Tyr8 residues. GnRH-II is primarily localized on the hypothalamus’s hippocampus, midbrain, and distinct nuclei. This is also highly expressed in the periphery, particularly in the kidneys, prostate, ovaries, and bone marrow. GnRH-II has different effects from GnRH-I, and it functions as a neuromodulator of sexual behavior. It may also regulate female reproductive behavior, permissively depending on the body’s energy and food intake [14,23]. In placental tissue and first-semester trophoblasts, GnRH-I and GnRH-II both cause a rise in human chorionic gonadotropin (HCG), with GnRH I producing a more significant increase than GnRH II [25]. Studies in humans have demonstrated that GnRH-I and its cognate receptor, GnRHR-I, exhibit significantly higher levels of expression in the hypothalamus and pituitary gland than GnRH-II and GnRHR-II, respectively. This differential expression pattern suggests a more prominent role for GnRH-I/GnRHR-I in regulating human reproductive function, making GnRH-I a primary therapeutic target for various reproductive disorders [23,26].

**Figure 1:**
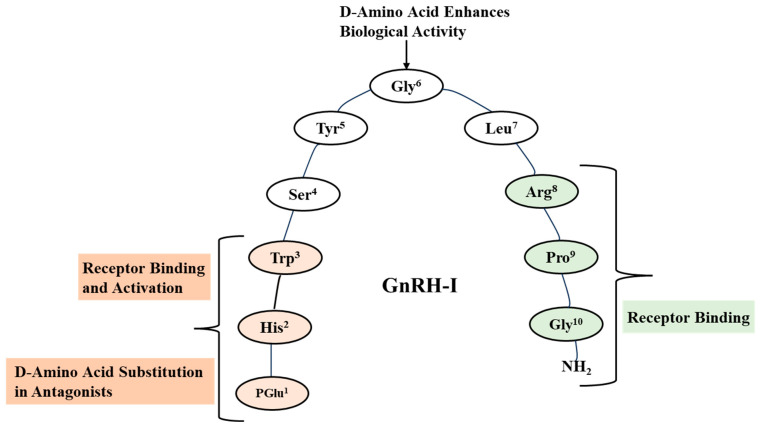
Schematic representation of mammalian GnRH in its folded conformation.

Both the carboxyl-terminal (amino acids 6–10) and amino-terminal (amino acids 1–6) of the GnRH-I peptide are essential for receptor binding. The amino terminal predominantly mediates receptor activation, while the carboxy-terminal, particularly Arg8, is responsible for high-affinity binding [1,18]. The specific amino acids at positions 1, 2, 3, 6, and 10 are crucial for the biological activity of the GnRH-I peptide. The modification to pyroglutamic acid at position 1(Pyr1) provides resistance to degradation by aminopeptidases, thereby extending the half-life of the peptide. Histidine at position 2 (His2) is critical in receptor binding. Substitutions at this position typically result in a loss of activity, indicating its importance in maintaining the correct conformation for receptor interaction. Position 3 is Tryptophan (Trp3), essential for receptor activation. Alterations here can significantly reduce the ability of GnRH to bind and activate its receptor. The amino acid glycine at position six (Gly6) contributes to the bending of the peptide into a characteristic horseshoe-like conformation, bringing both the termini closer for receptor interaction (Figure 1). Position 6 is also susceptible to enzymatic cleavage. Position 10 at the carboxy-terminal is occupied by the amino acid amidated glycine (Gly-NH2). The amidation of this glycine is essential for receptor binding and biological activity of GnRH-I [1,27].

Amino acid residues at positions 1, 6, and 10 within the native GnRH-I decapeptide are crucial for its three-dimensional structure. These conserved residues are essential for receptor binding within the anterior pituitary and subsequent release of gonadotropins LH and FSH. GnRH-I exhibits conformational flexibility, with a predominant β-II’ turn encompassing residues Tyr5-Gly6-Leu7-Arg8. This turn is stabilized by intramolecular interactions, particularly involving the Arg8 side chain. The amino acid at position 8 is crucial in ligand selectivity among different GnRH receptor subtypes. Arg at position 8 is essential for high-affinity binding to the mammalian pituitary GnRHR-I. The substitution of Arg8 leads to an extended peptide conformation, diminishing the prominence of folded conformers and consequently reducing biological activity. Notably, substituting the achiral glycine at position 6 with a D-amino acid enhances the β-II’ turn conformation, stabilizes the folded structure, and significantly increases (approximately 1000-fold) the binding affinity of Arg8-containing GnRH to the mammalian receptor. Furthermore, D-Gly6 substitution also reduces the metabolic clearance of GnRH-I [16,28].

### 4.2. GnRH Receptor: A Therapeutic Target for Reproductive Disorders

The hypothalamus and pituitary are the primary origin and target locations for GnRH. However, extra-pituitary and extra-hypothalamic GnRH receptors are also found in various reproductive organs, such as the myometrium, endometrium, and ovary. Through multiple mechanisms, including tissue remodeling, GnRH acts in a paracrine and autocrine fashion to control the proliferation and motility of cells in the endometrium during embryo implantation and the initial stages of pregnancy [1]. While traditionally recognized for its reproductive role, emerging evidence underscores additional functions of GnRH within the central nervous system (CNS). GnRHR expression extends beyond the reproductive HPG axis, with a widespread distribution encompassing the basal ganglia, basal forebrain, cerebral cortex, cerebellum, hippocampus, and spinal cord. This ubiquitous presence suggests a broader neuromodulatory role for GnRH in regulating emotions, motor function, cognition, and memory [13,29]. Numerous clinical disorders have been linked to disruptions in the GnRH-GnRHR signaling pathway. These include reproductive disorders, reproductive hormonal dependent oncological, and neurodegenerative diseases. Numerous investigations have shown the presence of GnRH and its corresponding receptor in a range of human malignant tumors that arise from the urogenital tract, such as endometrium, ovaries, bladder, and prostate malignancies. Moreover, GnRH receptor expression has also been documented in breast cancer, as well as in non-reproductive malignancies such as pancreatic cancer and glioblastoma. These outcomes suggest the autocrine function of the GnRH system in controlling vital cellular mechanisms like cell cycle progression, cell proliferation, and programmed cell death (apoptosis) in these tumor microenvironments [30,31,32].

## 5. GnRH Analogs

The first synthetic GnRH analog developed in 1985 was leuprolide, a GnRH agonist [33]. The development of synthetic GnRH analogs, such as leuprolide and buserelin, marked a significant advancement in controlling reproductive hormone release. These analogs feature specific modifications at positions 6 and 10 of the native GnRH-I structure, which improve their stability, receptor-binding affinity, and potency. At position 6, leuprolide incorporates D-leucine (D-Leu), while buserelin uses D-serine (D-Ser). Replacing naturally occurring L-amino acids with D-amino acids makes these analogs more resistant to enzymatic degradation. Most proteolytic enzymes do not readily recognize or cleave the D-amino acids.

Additionally, these changes increase the molecule’s hydrophobicity, enhancing its interaction with the GnRH receptor. At position 10, leuprolide and buserelin have an ethyl amide group instead of the natural glycine-amide. This modification protects the C-terminal end from enzymatic attack, further improving stability. Adding the ethylamine also increases the hydrophobicity, contributing to stronger receptor binding. Together, these modifications result in GnRH agonists like leuprolide and buserelin having 50–100 times greater potencies than the native GnRH-I [34].

Their ability to modulate the HPG axis has led to diverse clinical applications. GnRH agonists may be used to treat several hormone-dependent cancers and neurological diseases, as well as to regulate the synthesis of gonadotropin in the context of assisted reproductive technologies (ART) [35]. GnRH agonists exert pharmacological effects by binding to and activating the pituitary GnRH receptor. This initial stimulation leads to a transient surge in gonadotropin secretion. However, prolonged exposure to GnRH agonists results in receptor downregulation and desensitization, leading to a paradoxical suppression of gonadotropin release. This phenomenon, known as the “flare-up” effect, is followed by a hypogonadal state characterized by reduced sex steroid production. The hypogonadal state induced by GnRH agonists has been exploited therapeutically in managing hormone-dependent tumors, including prostate and breast cancer. In prostate and breast cancer, where sex steroids often drive tumor growth, GnRH agonists can effectively cease gonadotropin release and consequently suppress tumor progression by reducing the serum concentration of testosterone or estrogen to castrate levels [36,37]. In reproductive medicine, GnRH agonists play a pivotal role in controlled ovarian stimulation, facilitating the retrieval of multiple mature oocytes for ART [1,35].

Additionally, they have demonstrated potential in preserving ovarian function in young cancer patients undergoing chemotherapy or radiation. Due to this treatment, such young female patients experience gonadotoxicity, along with premature ovarian insufficiency (POI), infertility, and sexual dysfunction [38,39,40]. Beyond their oncological and reproductive applications, GnRH agonists have demonstrated therapeutic benefits in conditions associated with excessive estrogen production, like adenomyosis, uterine fibroids, and endometriosis. By suppressing estrogen synthesis, these agents can alleviate symptoms and improve patient quality of life [24,41,42,43]. Up to now, several GnRH agonists, including goserelin, triptorelin, and leuprolide, have been approved by the regulatory authorities and reached the market [43,44,45]. Despite their therapeutic potential, GnRH agonists are not without limitations. One notable drawback is the “flare effect”, a transient increase in gonadotropin secretion that can exacerbate symptoms in patients with estrogen-dependent conditions before the onset of downregulation. This effect is particularly relevant in endometriosis and uterine fibroids [46,47]. Additionally, prolonged usage of GnRH agonists has been associated with several hypogonadal adverse effects, including mood swings, vaginal dryness, decreased bone mineral density (BMD), and vasomotor symptoms [48,49]. Cardiovascular risk factors, consisting of lowered high-density lipoprotein cholesterol and elevated low-density lipoprotein cholesterol, have also been reported higher with GnRH agonists [50,51].

## 6. GnRH Peptide Antagonists

The first-generation antagonists such as NaI1-Arg6 ([Ac-D-2Nal1, D-Fpa2, D-Trp3, and D-Arg6] GnRH) were designed by substitution of the native amino acids with aromatic amino acid residues at positions 1, 2, 3, and 6 of GnRH-I. These compounds showed complete inhibition of ovulation and increased resistance to proteolysis. However, they had very poor water solubility and propensity to form gels. Incorporating D-alanine (D-Ala10) at position 10 generated newer antagonists with similar hydrophobicity only. This trend of higher hydrophobicity was altered by incorporating essential D-amino acids such as D-Arg at position 6. This substitution resulted in potent analogs with increased hydrophilicity, mitigating their tendency to form gels. Furthermore, D-Arg6 analogs exhibited a significantly prolonged duration of action. This prolonged duration of action was attributed to a “hydrophilic depot effect”, hypothesized to arise from electrostatic interactions between the positively charged D-Arg6 group and the negatively charged phospholipid cell membranes [16,52,53,54].

Based on these findings, detirelix, a potent and long-acting GnRH antagonist with the sequence [Ac-DNall, DCpa2, DTrp3, D-hArg (Et2)6, D-Ala10] GnRH was rationally designed to incorporate the D-hArg(Et2)6 moiety, enabling both electrostatic interactions and enhanced hydrophobic interactions with the lipid bilayer [54,55] evaluated the efficacy of detirelix in six healthy women with regular menstrual cycles. A regimen of 5 mg of detirelix was administered subcutaneously (SC) in the anterior abdominal wall every other day for 27 days. The study demonstrated that chronic administration of detirelix rapidly suppresses the hypothalamic–pituitary–ovarian axis, effectively inhibiting follicular development and ovulation. The mean serum levels of LH, estrogen, and progesterone were significantly lower during detirelix treatment than untreated controls. LH suppression was more pronounced and rapid than that of FSH. After the first injection, LH levels were suppressed within 2 h, with maximal suppression (74 ± 2%) observed by 8 h. In contrast, FSH levels after the first injection were suppressed by a maximum of 26 ± 3% within 8 h. No LH surge was observed during treatment. The mean estrogen levels also reduced significantly, from 165 ± 15 to 70 ± 11 pmol/L within 24 h of the first injection, and remained below 128 pmol/L throughout the treatment period [56]. GnRH antagonists containing D-Arg or other basic side chains at position 6, including detirelix, have been shown to induce histamine release from mast cells, limiting their clinical applicability. However, the rapid and reversible pituitary gonadal suppression achieved with detirelix highlights the potential therapeutic value of GnRH antagonists in steroid-dependent disorders. It emphasizes the need for continued development of safe and clinically effective antagonists [55,57].

**Figure 2:**
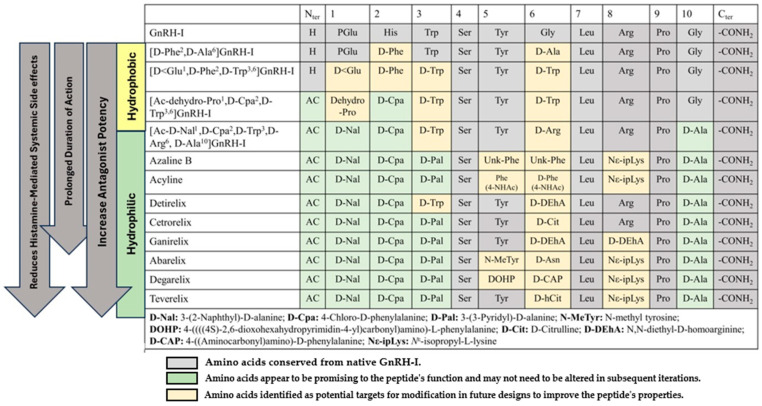
The evolution of the structure of GnRHs [16,54,55].

A few modifications have been explored to counteract these severe anaphylactoid reactions brought on by the rapid release of histamine. The primary one was the introduction of D-amino acid substitutes with unnatural side chains at the first three positions in native decapeptide. The most commonly used substitutes are N-acetyl-D-(b-naphthyl) alanine for the first position, D-(4-chloro) phenylalanine for the second, and D-(2-pyridyl) alanine or D-Trp for the third one. Neutral D-ureidoalkyl amino acids, which often carry chains containing amide, urea, or guanidine substituents such as D-citrulline, modify the amino acid at position 6. Another replacement aims to improve the solubilities and hydrogen bond-forming capacities of the GnRH peptide antagonist through alteration to the side chain in the eighth amino acid. Further modification includes substituting D-alaninamide with the C-terminal glycinamide. This change led to the development of third-generation antagonists with more excellent water solubility and substantial antagonistic activity without any edematogenic effects [34,53,55]. Figure 2 summarizes the evolutionary progression of amino acid sequences from first-generation to third-generation GnRH peptide antagonists.

Cetrorelix became the first clinically viable third-generation GnRH peptide antagonist approved in 1999. It is clinically indicated for controlled ovarian stimulation (COS) in women undergoing IVF to prevent premature LH surges. Shortly after Cetrorelix, Ganirelix was developed and received FDA approval in 2000 as another GnRH peptide antagonist for ART. Abarelix was the first GnRH peptide antagonist approved in the U.S. in 2003 for treating advanced prostate cancer. Clinical studies demonstrated that Abarelix could reduce serum testosterone to castrate levels without a “flare effect” more rapidly than clinically approved GnRH agonists with or without an antiandrogen. The safety profile of Abarelix was also found to be comparable to that of GnRH agonists.

Nonetheless, it has been attributed to a higher risk of systemic allergic reactions with quick onset, necessitating a comprehensive risk management approach [58,59]. Unfortunately, clinical use of Abarelix was discontinued in the US in 2005, although it is still used in Germany and the Netherlands [60]. Degarelix was approved by the FDA in 2008 and the EMA in 2009 for advanced, hormone-dependent prostate cancer. Compared to other GnRH peptide antagonists, it suppresses testosterone more rapidly and effectively. Unlike previous GnRH peptide antagonists, Degarelix has not been known to cause any systemic anaphylactic reactions in prostate cancer patients. However, despite its efficacy, its clinical use may be limited. This could be because of the higher frequency of injections required (every month as opposed to 1-, 3-, 6-, and 12-month formulations with GnRH agonists) and a high (~40%) incidence of injection site reactions [61,62,63].

Antev Ltd. (London, UK) has recently developed Teverelix, a novel decapeptide GnRH antagonist. The objective is to improve treatments like Degarelix by offering a more favorable administration schedule (fewer injections due to extended action) and better local tolerability.

With a dose that results in the half-maximal response, Teverelix exhibits low histamine-releasing activity, minimal in vitro aggregation, and comparatively good water solubility.

Phase II loading dose-finding trials have shown positive outcomes. A global phase III trial involving up to 1500 advanced prostate cancer patients with elevated cardiovascular risk is now underway. This trial will compare Teverelix to the standard of care (GnRH agonist) in a 1:1 randomized design. The primary objective is to provide definitive evidence supporting Teverelix as the preferred treatment option for this patient population. The trial is expected to conclude by 2027 [64,65,66,67]. Table 1 summarizes the GnRH peptide antagonists that have been clinically evaluated until November 2024.

## 7. Comparative Analysis of GnRH Peptide Antagonists

The most promising third- and fourth-generation GnRH antagonists contain hydrophobic clusters of Ac-D-Nal-D-Cpa-D-Pal in the N-terminal and Pro-D-Ala in the C-terminal. The marketed synthetic peptide antagonists Abarelix, Cetrorelix, Ganirelix, Degarelix, and Teverelix, which are under clinical phase III, share highly similar structural formulas. Cetrorelix, Ganirelix, and Teverelix differ by two amino acids at positions 6 and 8 of the peptide chain. On the other hand, Abarelix, Degarelix, and Teverelix differ by two amino acids at positions 5 and 6 of the peptide chain (Figure 2). The following sections will explore and compare their pharmacokinetic properties, efficacy, safety profiles, and formulation strategies.

### 7.1. Comparative Formulation Behaviour of GnRH Peptide Antagonists

Until 2024, Abarelix, Ganirelix, Cetrorelix, and Degarelix have been granted marketing authorization [68,69]. They share the same mechanism of action but differ in their formulations and administration routes to achieve their therapeutic effects.

Ganirelix and Cetrorelix are used in medical practice for the inhibition of premature LH surges in women undergoing COS for ART. Ganirelix is formulated as a ready-to-use pre-filled syringe (PFS). Each PFS contains 0.25 mg of Ganirelix acetate in 0.5 mL aqueous solution, glacial acetic acid, mannitol, and water for injection. On the other hand, Cetrorelix exhibits a propensity for aggregation and gel formation. Lyophilization from an acidic aqueous solution effectively mitigates these issues [70,71].

Consequently, Cetrorelix is marketed as a sterile lyophilized powder for SC injection. Reconstitution with sterile water for injection (SWFI) yields a solution with a pH of 4.0–6.0. Each vial contains 0.25 mg Cetrorelix (equivalent to 0.26–0.27 mg Cetrorelix acetate) and mannitol as a lyoprotectant [72,73,74].

Abarelix and Degarelix are clinically indicated for the treatment of advanced prostate cancer. Both the GnRH antagonists are available as a sterile, dry powder that, upon reconstitution, forms depots, facilitating loading of high drug concentrations and providing extended release of the drug. Abarelix is formulated as a suspension for injection containing Abarelix acetate complexed with carboxymethyl cellulose (Abarelix–CMC). In the Abarelix–CMC complex, CMC acts as a carrier molecule that, upon administration, enables the continuous release of Abarelix for a prolonged period of one month. The marketed formulation contains a 113 mg anhydrous-free base Abarelix in single-dose vials. The sterile, white to off-white lyophilized powder of the Abarelix–CMC complex is reconstituted with 2.2 mL of 0.9% sodium chloride solution. Each 2 mL intramuscular (IM) injection delivers a 100 mg dose of Abarelix at a pH of 5 ± 1 [8,75]. Degarelix for injection is also a sterile lyophilized powder that contains Degarelix and mannitol. The lyophilized powder is reconstituted with water for injection.

In this case, reconstitution takes 15 min and should be administered within 45 min. As on standing at ambient temperature, the reconstituted solution self-associates and starts forming a non-suspendable turbid solution after more than 4 h. Finally, it forms a viscous gel within a few minutes after administration. Hence, the reconstituted solution should be used within an hour of reconstitution at ambient temperature. SC administration of Degarelix immediately forms a depot at the site of injection, and the drug is intended to be released from the depot for an extended time. The starting dose of 240 mg (120 mg/3 mL × 2) was followed by a maintenance dose of 80 mg (80 mg/4 mL) for one month’s dosing regimen. Each 120 mg drug product should be reconstituted with 3 mL of water for injections. For the maintenance dose, 80 mg drug product should be reconstituted with 4.2 mL of water for injection. It should not be reconstituted with bacteriostatic water for injection as it forms a precipitate [8,76].

### 7.2. Comparative Pharmacokinetics of GnRH Peptide Antagonists

Table 2 outlines the pharmacokinetic behavior of clinically meaningful GnRH peptide antagonists. Abarelix and Teverelix are administered via the IM route. At the same time, other GnRH peptide antagonists, including Cetrorelix, Ganirelix, and Degarelix, are administered via the SC route. Broqua and team conducted a comparative study to evaluate the duration of LH suppression induced by Abarelix, Cetrorelix, and Degarelix after SC and intravenous (IV) injections to castrated rats at 200 µg/kg. Regardless of the administration route, Abarelix demonstrated a consistent 12-h suppression of the LH levels [77].

In contrast, significant disparities in the duration of LH suppression were observed between IV and SC administrations of Cetrorelix and Degarelix. While both agents exhibited a 12-h suppression following IV injection, their SC administration yielded more prolonged effects. Cetrorelix maintained maximal LH suppression for 2 days, whereas Degarelix extended this effect to 6 days [77].

Compared to Abarelix, the prolonged duration of action of Cetrorelix can be attributed to its enhanced capacity for intramolecular hydrogen bonding. Key structural modifications in Cetrorelix, including the incorporation of L-Arg and D-Cit residues at positions 4 and 6, respectively, as well as the demethylation of the amide at position 7 (Tyr residue), contribute to this increased hydrogen bonding potential. The substitution of amide and secondary amine groups with urea and guanidine moieties significantly enhances the hydrogen bonding capabilities. Additionally, removing the N-methyl group from the amide at position 7 increases the number of available hydrogen bond acceptors, further stabilizing the molecule’s structure [82].

Degarelix is administered subcutaneously in the abdominal region. The standard treatment involves a 240 mg initial dose followed by 80 mg maintenance doses every 28 days. This regimen effectively suppresses testosterone levels below castration levels in approximately 97% of patients for at least a year. The minimal effective dose of Degarelix for suppressing LH or testosterone levels was determined to be between 1 and 3 µg/kg, comparable to Abarelix. However, unlike Abarelix, Degarelix demonstrated a dose-dependent increase in the duration of action. While increasing the dose from 12.5 to 200 µg/kg did not enhance efficacy, it significantly prolonged LH suppression from 1 to 7 days. Compared to the preceded GnRH peptide antagonists, Degarelix demonstrates improved solubility and a significantly prolonged duration of action. This is hypothesized to arise from the increased hydrogen bonding potential, which likely influences solubility and receptor binding stability. However, the affinity of Degarelix for the GnRHR is comparable to that of Ganirelix, Abarelix, and Cetrorelix [14,83]. Its prolonged action is primarily attributed to sustained-release depot formation at the subcutaneous injection site. This depot formation is facilitated by Degarelix’s intrinsic tendency to gel at concentrations above ≈ 1 mg/mL. This in vivo gelling creates a depot immediately following subcutaneous administration.

In contrast, Cetrorelix and Ganirelix, administered at lower doses (0.25 mg/day), likely do not reach the concentration threshold required for gel formation and thus lack this sustained-release depot mechanism [84]. Degarelix exhibits concentration-dependent pharmacokinetics. The relative bioavailability of Degarelix was inversely proportional to the dose concentration. The estimated values for bioavailability from population pharmacokinetic modeling were approximately 60% and 40% for dose concentrations 20 mg/mL and 40 mg/mL, respectively. This is likely due to increased gel rigidity at higher concentrations, hindering drug release from the depot. However, the pharmacokinetic profile of Degarelix is consistent with “flip-flop” kinetics, where absorption is the rate-limiting step, influencing the apparent elimination rate [84,85,86,87].

Abarelix exhibits slow absorption following IM administration. A 100 mg dose resulted in peak plasma concentrations approximately 3 days post-injection. Treatment with Abarelix rapidly suppressed LH, testosterone, DHT, and prostate-specific antigen (PSA) levels. Within eight days, approximately 75% of patients achieved castrate testosterone levels (<5 ng/mL) without a testosterone surge. A depot formulation significantly improved Abarelix bioavailability, increasing the half-life from 5.3 h for a single injection to 13.2 days for the depot formulation. The depot formulation demonstrated a more significant suppression of testosterone, DHT, LH, and FSH than single injections. The mean inhibition rates for testosterone, DHT, LH, and FSH were 93.6%, 88.5%, 94.6%, and 71.2%, respectively, with the depot significantly higher than the single-injection group (testosterone: 76.5%, DHT: 65.2%, LH: 76.5%, and FSH: 33.6%) [14,83].

Cetrorelix and Ganirelix have similarly established their utility in COS for ART due to their ability to rapidly and reversibly suppress endogenous LH at low antagonist concentrations, while the suppression of the FSH level was not so pronounced. Two different treatment regimens are currently established for ovarian stimulation. The multiple-dose protocol involves daily SC administration of 0.25 mg Cetrorelix or Ganirelix from day 6 of gonadotropin treatment until hCG administration for ovulation induction. The single-dose protocol utilizes a 3 mg dose of Cetrorelix on day 7 of gonadotropin treatment, providing a four-day protection period. Additional 0.25 mg Cetrorelix doses may be administered if the ovulation induction criteria are not met within four days of the initial 3 mg dose. Comparative studies have demonstrated comparable efficacy and safety profiles for both regimens. Notably, only Cetrorelix is available in both single- and multiple-dose formulations. A minimal effective dose of 0.25 mg/day administered SC for both Cetrorelix and Ganirelix has been determined to be sufficient to prevent premature LH surges and ensure the development of high-quality oocytes. Both the peptide therapeutics are rapidly absorbed following SC injection, with a mean absolute bioavailability in healthy female subjects of 85% and 91%, respectively. The elimination half-life of Ganirelix after single-dose administration was about 13 h, which was longer than that of Cetrorelix (between 5 and 10 h) [14,88,89]. No significant differences in pharmacokinetic parameters were observed between repeated and single 0.25 mg doses of Cetrorelix and Ganirelix, as detailed in Table 2.

Teverelix, formulated as a TFA (trifluoroacetate) salt, forms a microcrystalline suspension at the concentration used for injection, allowing a small injection volume. It is speculated that the rapid removal of TFA post-injection enables the formation of a gel depot, inhibiting rapid aggregation of Teverelix. SC administration of Teverelix results in a prolonged half-life compared to IM injection. This is likely due to vascularization at the site of injection, the motion of the site of injection, and the fat content of the site of injection, as Teverelix potentially becomes trapped in fat cells and lipoprotein membranes. The small injection volume of Teverelix, typically 0.6 to 1.6 mL, covering the expected therapeutic range for Teverelix in various indications of 45 to 120 mg, contributes to its good local tolerability. SC and IM routes of administration of drugs are standard of care. Still, in the case of SC injections, the hypodermis’s extracellular matrix reduces the tissue space’s compliance to injected fluids, such that SC injections are generally limited to 1.5 mL and are associated with injection pain and adverse events at the injection site. Like Degarelix, Teverelix has been shown to suppress LH and FSH in a dose-dependent manner. SC administration of Teverelix TFA produced slightly lower Cmax and AUC compared to IM administration of the same dose, but the slow-release phase characteristics differ significantly. The Cmax of the slow-release phase was approximately four-fold higher for the IM dose compared to the same SC dose level. The slow-release phase tmax was also observed much earlier in the IM administration. This prolonged earlier exposure following IM administration is commensurate with the increased duration of action on the inhibition of serum FSH, LH, and testosterone. Marked slow-release differences in tmax (IM = 4 days; SC = 21 days), Cmax (IM = 12.4 ng/mL; SC = 3.2 ng/mL), and half-life (IM = 15 days; SC = 78 days) allow tailoring dosing regimens that will deliver the appropriate amount of Teverelix over the relevant time frame to achieve the pharmacologic effect required for a variety of indications. Teverelix TFA has the potential to be a safer, better alternative to marketed products and, mainly where injection is the preferred mode of delivery, could meet unmet clinical needs in multiple indications [64,67].

### 7.3. Comparative Clinical Efficacy and Safety Profile of GnRH Peptide Antagonists

GnRH peptide antagonists, such as Abarelix, Cetrorelix, Ganirelix, Degarelix, and Teverelix, demonstrate significant efficacy in suppressing gonadotropin release. This suppression is critical for treating hormone-dependent conditions such as prostate cancer, endometriosis, and uterine fibroids and facilitating COS in ART. Below is a detailed discussion of their efficacy based on clinical studies and real-world applications.

Cetrorelix is extensively used in ART to prevent premature LH surges during COS, ensuring proper timing for oocyte retrieval. Binding to pituitary GnRH receptors rapidly and reversibly suppresses gonadotropin secretion, leading to controlled follicular development. In a randomized trial by Olivennes et al. (2000), Cetrorelix showed comparable pregnancy rates to long GnRH agonist protocols (36% vs. 38%) [90]. On the other hand, other studies show that Cetrorelix significantly lowers the incidence of OHSS (ovarian hyperstimulation syndrome) compared to GnRH agonists [91,92]. The use of Cetrorelix allows for shorter treatment durations, which can improve patient adherence and satisfaction.

Lower gonadotropin doses are also required, reducing the overall treatment burden and associated costs [93]. All this makes it a practical and patient-friendly alternative to agonist protocols. Its efficacy in suppressing premature LH surges has been pivotal in ART, particularly for high-risk patients prone to OHSS.

Ganirelix is another GnRH antagonist used in ART, offering similar efficacy to Cetrorelix. Its rapid action prevents premature ovulation, ensuring follicular maturity and timing for oocyte retrieval. A meta-analysis by Al-Inany and the team confirmed that Ganirelix-based protocols result in pregnancy and live birth rates equivalent to agonist protocols [74,94]. Ganirelix, a GnRH antagonist, effectively prevents premature LH surges during ART, ensuring optimal follicular maturity for oocyte retrieval. Its rapid absorption and high bioavailability make it a preferred choice in ART protocols. Ganirelix effectively inhibits premature LH surges, which can disrupt follicular maturation and lead to cycle cancellations [95]. A daily dose of 0.25 mg has been identified as optimal for maintaining high ongoing pregnancy rates [96]. Studies show that Ganilever, a new formulation of Ganirelix, demonstrates comparable outcomes to Orgalutran, another GnRH antagonist, regarding oocyte retrieval and embryo quality [97]. The use of GnRH antagonists like Ganirelix has been associated with reduced ovarian hyperstimulation syndrome compared to long GnRH agonist protocols [96]. Ganirelix’s rapid action allows for timely oocyte retrieval, enhancing the chances of successful fertilization and pregnancy [96,98]. The protocol’s flexibility and reduced patient burden make it a favorable option in ART settings [96]. While Ganirelix shows significant benefits, some studies suggest that long GnRH agonist protocols may yield higher pregnancy rates in specific populations, indicating a need for further research to optimize ART outcomes [96,99]. Thus, Ganirelix’s consistent cycle control and comparable reproductive outcomes make it a standard choice in ART. In high-risk patients, Ganirelix significantly reduces the risk of OHSS.

Abarelix effectively reduces both LH and FSH secretion, a result that is noticeably greater than that of Cetrorelix. Because of this consequence, Abarelix cannot be used in COS/ART procedures where it is not desired for FSH levels to drop. In vitro, however, it was demonstrated that FSH could promote the growth of human androgen-resistant prostate cancer. Abarelix was the only GnRH antagonist to be released into the market as an antitumor agent in 2003, and it was highly effective in treating prostate cancer [83]. Unfortunately, clinical use of Abarelix was discontinued in the US due to allergic reactions in 2005 [60,100].

ADT with long-acting luteinizing hormone-releasing hormone (LHRH) agonists is the principal systemic treatment for hormone-sensitive prostate cancer (PC). Degarelix is the first potent GnRH antagonist primarily used in prostate cancer. Previously developed Cetrorelix and Ganirelix, which have a shorter half-life, require daily SC injections, making them unsuitable for long-term prostate cancer treatment, which requires continuous androgen suppression [101,102]. Pivotal clinical trials, including the study by Klotz et al. (2008), demonstrated that Degarelix consistently maintained testosterone levels below castration thresholds (<50 ng/dL) in over 96% of patients for up to one year [62]. It achieves rapid testosterone suppression within 1–3 days of administration, avoiding the testosterone surge associated with GnRH agonists (tumor flare). This rapid suppression is particularly beneficial in advanced or metastatic prostate cancer, alleviating symptoms and preventing complications like spinal cord compression [103,104]. Degarelix achieves a rapid decline in testosterone levels, significantly outperforming leuprolide in the initial treatment phase [103,105]. Additionally, it reduced prostate-specific antigen (PSA) levels more effectively in the first month of treatment, which is a key indicator of prostate cancer progression [103]. These findings highlight its superiority in the acute phase of treatment.

Teverelix has demonstrated significant clinical efficacy and safety in the treatment of advanced prostate cancer and benign prostatic hyperplasia (BPH) in multiple phase II clinical trials. In an adaptive phase II, open-label, multicenter trial, patients (n = 41) with advanced prostate cancer received a 180 mg SC + 180 mg IM dose of Teverelix at a single visit, followed by 6-weekly SC maintenance doses of 180 mg up to Day 168. A rapid (within 2 days) and sustained suppression of testosterone, PSA, FSH, and LH to castration levels was observed. The most common adverse effects were injection site induration, injection site erythema, and hot flush. Most injection site reactions were Grade 1. Overall, the Teverelix DP at the administered dose was generally well tolerated. However, the castration rates were not maintained to Day 42 with this dosage regimen and required further optimization [65].

Another phase II, randomized, double-blind, placebo-controlled study evaluated the efficacy and safety of Teverelix in patients with symptomatic BPH. After a 4-week single-blind placebo run-in, patients were randomized to receive Teverelix at 60 mg (n = 41) or a placebo (n = 40) subcutaneously on days 1 and 3. At the end of the treatment period (Week 16), Teverelix significantly reduced the International Prostate Symptom Score (IPSS). By Week 2, Teverelix showed a mean IPSS reduction of 13.0%, reaching 34.5% by Week 16. Clinically relevant IPSS reductions were observed in 44% of Teverelix patients by Week 2, increasing to 83% by Week 12. Teverelix also achieved a significant 11% reduction in prostate volume within 4 weeks, improved the maximum urinary flow rate, and enhanced the quality of life (QoL) scores [66].

These findings suggest that Teverelix is a promising therapeutic option for both advanced prostate cancer and BPH. Further studies are warranted to evaluate its long-term efficacy and safety profile.

## 8. Safety Profiles of Various GnRH Peptide Antagonists

GnRH antagonists with D-arginine or D-Lysine at position 6 and a cluster of hydrophobic amino acids at the N-terminus have been associated with histamine release and subsequent adverse reactions such as facial and extremity edema and cutaneous anaphylactoid-like reactions. These side effects were particularly evident with first-generation molecules. Abarelix was the first GnRH antagonist clinically approved for the treatment of prostate cancer. However, numerous drawbacks hampered its clinical use. The most frequently reported adverse events included decreased libido or symptoms of headaches, fever, hot flushes, and upper respiratory tract disorders (bronchitis). Because of this adverse effect, in 2003, the FDA approved the use of Abarelix (Plenaxix) only for patients with advanced, symptomatic prostate cancer who could not have or refused other treatments. Additionally, 1.1% of treated individuals experienced an acute systemic allergic reaction within 8 min of receiving an Abarelix injection [14,71]. As a result, in 2005, Praecis Pharmaceuticals voluntarily shut down the use of Abarelix in the United States, and it was no longer used for cancer therapy [83].

Koechling et al. (2010) compared the histamine-releasing potential of various GnRH antagonists using an ex vivo human skin sample model. He observed significant differences between antagonists, with Degarelix having the lowest capacity (>30 µg/mL), followed by Ganirelix (>10 µg/mL), and Abarelix and Cetrorelix having the highest capacity (>3 µg/mL). Teverelix TFA also exhibits weak histamine-releasing properties in vitro (EC50 = 81 µg/mL) [64,106]. Broqua et al. 2002 reported the relative order of potency in stimulating histamine release in vitro was Cetrorelix (EC50 = 1.3 µg/mL) > Ganirelix (EC50 = 11 µg/mL) > Abarelix (EC50 = 100 µg/mL) > Degarelix (EC50 = 170 µg/mL) [77]. Teverelix and Cetrorelix differ by only two amino acids at positions 6 and 8. Teverelix contains D-homocitrulline, while Cetrorelix contains D-citrulline at the 6th position, which have almost identical residues and differ only by one carbon in their side chain. Although, there exists a significant difference in histamine-releasing properties. Cetrorelix exhibits a histamine-releasing potency with EC50 = 1.3 µg/mL, while Teverelix exhibits a histamine-release potency with EC50 = 81 µg/mL.

Due to higher histamine-releasing properties than Abarelix and Degarelix, Cetrorelix and Ganirelix cannot be clinically indicated as antineoplastic agents required to be administered at higher doses compared to ART treatment. However, the administration of Cetrorelix and Ganirelix at lower doses can result in modest local responses such as pain, edema, and erythema when injected subcutaneously. Usually, these reactions are temporary and do not result in discontinuation [107,108]. Overall, Cetrorelix and Ganirelix are considered safe for short-term use in ART, with infrequent and mild adverse effects.

Degarelix has demonstrated a lower risk of cardiovascular events compared to GnRH agonists like leuprolide. This is particularly significant in prostate cancer patients, many of whom have pre-existing cardiovascular conditions [109,110]. Prolonged testosterone suppression can lead to decreased bone mineral density (BMD) and an increased risk of fractures in patients. This shared risk with all ADT agents requires monitoring [111,112]. Degarelix is generally well tolerated and offers a favorable safety profile, particularly for patients at high cardiovascular risk. However, its injection site reactions are more frequent compared to other antagonists. 

## 9. Conclusions

In conclusion, GnRH analogs, both agonists and antagonists, have emerged as pivotal therapeutic modalities for a spectrum of reproductive disorders, including infertility treatments and hormone-dependent cancers. While GnRH agonists have been widely employed, GnRH antagonists have gained significant attention due to their rapid and reversible suppression of gonadotropins without the initial surge and flare-up effect associated with GnRH agonists. The structural modifications introduced in GnRH antagonists, particularly at positions 6 and 8, have been instrumental in optimizing their pharmacological properties.

However, despite sharing structural similarities, GnRH antagonists exhibit diverse pharmacokinetic and pharmacodynamic profiles. For instance, with their inherent gel formation properties, Abarelix and Degarelix offer advantages in managing prostate cancer. Conversely, Cetrorelix and Ganirelix, with their distinct suppression profiles of LH and FSH, are well-suited for applications in assisted reproductive technologies. While these peptide-based antagonists have demonstrated clinical efficacy, limitations such as short half-lives and the need for parenteral administration hinder their broader application. To overcome these challenges, future research should prioritize the development of novel GnRH antagonists with improved pharmacokinetic and pharmacodynamic properties.

A key area for advancement lies in the exploration of non-natural amino acids, particularly at positions 6 and 8 of the GnRH molecule. By systematically investigating structure–activity relationships, researchers can identify modifications that enhance the receptor binding affinity, increase metabolic stability, and prolong the duration of action. Furthermore, the development of sustained-release formulations, such as implants or long-acting injectables, can significantly improve the convenience and adherence to therapy for patients.

While oral GnRH small molecule antagonists offer a promising avenue, optimizing the delivery of peptide-based antagonists via oral routes should not be neglected. Strategies such as peptide conjugation, encapsulation in nanoparticles, and the use of permeation enhancers warrant further investigation to enable effective oral delivery and improve patient compliance.

By addressing these critical areas, researchers can pave the way for the development of next-generation GnRH peptide antagonists with enhanced therapeutic profiles, including improved potency, selectivity, and bioavailability. These advancements will ultimately expand the clinical utility of GnRH antagonists and provide more effective treatment options for a wider range of reproductive and endocrine disorders.

## Figures and Tables

**Table 1:** Summary of GnRH peptide antagonists clinically tested until November 2024.

Name of Dug.	Brand	Approval Year	Approved Clinical Indication	Amino Acid Sequence	Molecular Weight
Detirelix	Discontinued after Phase 1 clinical study Indications: Contraception			Ac-D-2Nal-D-Phe(4-Cl)-D-Trp-Ser-Tyr-D-hArg(Et,Et)-Leu-Arg-Pro-D-Ala-NH2	1538.2
Ganirelix	Antagon	1999	Recommended in a clinical setting to prevent early LH surges in patients receiving controlled ovarian stimulation	Ac-D-2Nal-D-Phe(4-Cl)-D-3Pal-Ser-Tyr-D-hArg(Et,Et)-Leu-hArg(Et,Et)-Pro-D-Ala-NH2	1570.3
Cetrorelix	Cetrotide	2000	Clinically demonstrated to prevent early LH surges in females receiving regulated ovarian stimulation	Ac-D-2Nal-D-Phe(4-Cl)-D-3Pal-Ser-Tyr-D-Cit-Leu-Arg-Pro-D-Ala-NH2	1431.0
Abarelix	Plenaxis	2003	Clinically advised for the palliative care of males with advanced symptomatic prostate cancer who reject surgical castration and for whom LHRH agonist therapy is inappropriate because they have one or more of the following symptoms: (1) Likelihood of neurological impairment from metastases; (2) blockage of the ureter or bladder outlet because of local invasion or metastatic illness; or (3) excruciating bone pain from skeletal metastases that does not go away when using narcotic analgesics.	Ac-D-2Nal-D-Phe(4-Cl)-D-3Pal-Ser-N(Me)Tyr-D-Asn-Leu-Lys(iPr)-Pro-D-Ala-NH2	1416.1
Degarelix	Firmagon	2008	This medication is clinically advised for advanced prostate cancer.	Ac-D-2Nal-D-Phe(4-Cl)-D-3Pal-Ser-Phe(4-S-dihydroorotamido)-D-Phe(4-ureido)-Leu-Lys(iPr)-Pro-D-Ala-NH2	1632.3
Teverelix	Till Aug 2024, Under clinical development by Antev and currently in Phase 2 for Metastatic Prostate Cancer.			Ac-D-2Nal-D-Phe(4-Cl)-D-3Pal-Ser-Tyr-D-hCit-Leu-Lys(iPr)-Pro-D-Ala-NH2	1459.1
Prazarelix	Under clinical development			Ac-D2Nal-D4Cpa-D3Pal-Ser-4Aph(Ac)-D4Aph(Ac)-Leu-ILys-Pro-DAla-NH2	1613.3
Iturelix	Discontinued in Phase 2 clinical study Indications: Endometriosis, Infertility in Female			Ac-D-2Nal-D-Phe(4-Cl)-D-3Pal-Ser-Lys(nicotinoyl)(nicotinoyl)-D-Lys(nicotinoyl)(nicotinoyl)-Leu-Lys(iPr)-Pro-D-Ala-NH2	1591.3
Acyline	Discontinued in Phase 3 clinical study Indications: Contraception, Hypogonadism, Prostatic Cancer			Ac-D-2Nal-D-Phe(4-Cl)-D-3Pal-Ser-Phe(4-NHAc)-D-Phe(4-NHAc)-Leu-Lys(iPr)-Pro-D-Ala-NH2	1533.2
Ozarelix	Discontinued in Phase 3 clinical study Indications: Alzheimer’s Disease, Endometriosis, Lower Urinary Tract Symptoms			Ac-D-2Nal-D-Phe(4-Cl)-D-3Pal-Ser-N(Me)Tyr-D-hCit-Nle-Arg-Pro-D-Ala-NH2	1459.1
Ramorelix	Discontinued in Phase 1 clinical study Indications: Leiomyoma, Neoplasms, Prostatic Cancer			Ac-D-2Nal-D-Phe(4-Cl)-D-Trp-Ser-Tyr-D-Ser(but)-Leu-Arg-Pro-azgly-NH2	1532.1

**Table 2:** Pharmacokinetic parameters for marketed and investigational GnRH peptide antagonists.

	Type of Formulation	Dosage	n	Cmax (ng/mL)	tmax	t1/2	AUC0-∞	Reference
Abarelix	Depot Suspension	Single Dose Regimen: 100 mg is administered IM.	14	43.4 ± 32.3	3.0 ± 2.9 (days)	13.2 ± 3.2 (days)	500 ± 96 (ng.day/mL)	[78]
Cetrorelix	Injectable Solution	Single Dose Regimen: 3 mg is administered SC on day 7 of controlled ovarian stimulation.	12	28.5 (22.5–36.2)	1.5 (0.5–2) h	62.8 (38.2–108) h	536 (451–636) (ng.h/mL)	[79]
		Multiple Dosage Regimen: 0.25 mg is administered SC once daily during the early- to mid-follicular phase on either stimulation day 5 or day 6 until the day of hCG administration.	12	6.42 (5.18–7.96)	1.0 (0.5–2) h	20.6 (4.1–179.3) h	44.5 (36.7–54.2) (ng.h/mL)	
Ganirelix	Injectable Solution	Single Dose Regimen: 0.25 mg is administered SC.	15	14.8 (3.2)	1.1 (0.3) h	12.8 (4.3) h	96 (12) (ng.h/mL)	[80]
		Multiple Dosage Regimen: 0.25 mg is administered SC once daily from the mid to late follicular phase until the day of hCG administration.		11.2 (2.4)	1.1 (0.2) h	16.2 (1.6) h	77.1 (9.8) (ng.h/mL)	
Degarelix	Depot Injectable Solution	240 mg given as two SC injections of 120 mg.		26.2 (CV 83%)			1054 (CV 35%) (ng.day/mL)	[81]
Teverelix	Depot Microcrystalline Suspension	Teverelix trifluoroacetate 90 mg IM Single Dose	12	19.30 ± 4.136	1.520 (1.00–1.53) h	388.8 ± 196.5 h	5062 ± 1292 (ng.h/mL)	[64]
		Teverelix trifluoroacetate 60 mg SC Single Dose		7.238 ± 1.535	2.500 (1.93–3.00) h	1585 ± 797.8 h	2638 ± 586.5 (ng.h/mL)	
		Teverelix trifluoroacetate 90 mg SC Single Dose		12.57 ± 2.832	2.510 (1.50–3.98) h	2009 ± 791.6 h	4257 ± 1379 (ng.h/mL)	
		Teverelix trifluoroacetate 120 mg SC Single Dose		16.34 ± 3.689	2.570 (1.00–3.05) h	1880 ± 674.9 h	5051 ± 1601 (ng.h/mL)	

## List of Abbreviations

ADT	Androgen Deprivation Therapy
ART	Assisted Reproductive Technologies
CaMKII	Calmodulin-Dependent Protein Kinase II
cAMP	Cyclic Adenosine Monophosphate
CNS	Central Nervous System
CREB	cAMP Response Element-Binding protein
DAG	Diacylglycerol
ERK1/2	Extracellular signal-regulated Kinase 1 and 2
FSH	Follicle-Stimulating Hormone
Gαs	Gs alpha subunit
GnRH	Gonadotropin-Releasing Hormone
GnRHR	Gonadotropin-Releasing Hormone Receptor
GPCR	G protein-Coupled Receptor
HCG	Human Chorionic Gonadotropin
HPG	Hypothalamic–Pituitary–Gonadal
IP3	Inositol Trisphosphate
IVF	In Vitro Fertilization
JNK	Jun-N-terminal Kinase
LH	Luteinizing Hormone
MAPKs	Mitogen-Activated Protein Kinases
NFAT	Nuclear Factor of an Activated T cell
PCOS	Polycystic Ovary Syndrome
PIP2	Phosphatidylinositol 4,5-bisphosphate
PKA	Protein Kinase A
PKC	Protein Kinase C
PLCβ	Phospholipase Cβ
